# Pharmacogenetic-guided cannabis usage in the community pharmacy: evaluation of a pilot program

**DOI:** 10.1186/s42238-020-00033-1

**Published:** 2020-09-01

**Authors:** John Papastergiou, Wilson Li, Carly Sterling, Bart van den Bemt

**Affiliations:** 1 Leslie Dan Faculty of Pharmacy, University of Toronto, Toronto, Canada; 2 School of Pharmacy, University of Waterloo, Kitchener, ON N2G 1C5 Canada; 3 Shoppers Drug Mart, Toronto, ON M4J 1L2 Canada; 4 Sint Maartenskliniek, Nijmegen, The Netherlands; 5 Radboud University Medical Center, Nijmegen, The Netherlands

**Keywords:** Pharmacogenomics, Cannabis, Pharmacy practice, Point-of-care, Community pharmacy

## Abstract

**Background:**

Pharmacists possess a skillset suited to provide evidence-based guidance to current and potential users of cannabis. Clinical pharmacogenomics research has made significant progress in defining which genetic variations are important for influencing inter-patient variability in response to cannabis. This study aims to evaluate the practicality and impact of pharmacogenetic testing in the community pharmacy to help guide in the safe use of cannabis.

**Methods:**

The pilot program was designed as open-label, non-randomized, and observational. Two busy, urban community pharmacies, operating under the brand Shoppers Drug Mart, in Toronto, Ontario, Canada offered pharmacogenomic testing to cannabis users as part of their professional services program over a period of 2 months. Eligible patients received buccal swabs using a DNA cheek swab kit. De-identified, barcoded samples were then sent by regular mail to an off-site CLIA-certified laboratory for analysis in Mississauga, Canada. A pharmacogenetic testing platform from Lobo Genetics® was utilized for translation of participants’ DNA with respect to CYP2C9, AKT1 and COMT genetic polymorphisms. Following genomic data translation, personalized, evidence-based recommendations were generated. Pharmacists provided a cannabis pharmacogenetic consultation to patients via telephone or in-person.

**Results:**

Twenty patients enrolled in the study. Pharmacogenetic screening identified 95% as having the CYP2C9*1/*1 genotype (suggesting normal THC metabolism); 35 and 25% had AKT1 genotypes suggesting intermediate risk (C/T genotype) or high risk (C/C genotype), respectively, for cannabis-induced psychosis; and 45 and 10% had COMT genotypes suggesting intermediate risk (Val/Met genotype) or high risk (Val/Val genotype), respectively for cannabis-induced neurocognitive impairment.

After the pharmacogenetic consultation, 65% of patients reported an increased comfort level in choosing a specific strength/strain of cannabis for use in the future; 75% considered the consultation of high value providing information potentially vital to their health and wellbeing.

**Conclusion:**

Although the study did not find any CYP2C9 variants associated with highly diminished THC metabolism, most of these patients do carry genetic variants that may potentially predispose them to the development of psychosis and memory impairment. Similar initiatives can potentially improve patient safety and empower individuals to make informed decisions about cannabis use and possible complications.

## Introduction

As cannabis use becomes more prevalent, it is critical for the public to be educated on its potential therapeutic benefits, but more importantly, on the associated risks and variability in individual responses (Government of Canada n.d.-a; Government of Canada n.d.-b). Users of cannabis often select their product of choice through trial-and-error, which often can lead to adverse outcomes. Pharmacists are highly accessible health care professionals who are trained to provide evidence-based guidance to current and potential users of cannabis (Dattani and Mohr 2019). Historically, pharmacists’ involvement in cannabis education has been limited by their training and cannabis-related experience. This is further made difficult from the lack of standardization of cannabis products and availability of tools to predict individual response and tolerance from genetic variability (Hryhorowicz et al. 2018). In recent years, clinical pharmacogenetic research has made significant progress in defining which genetic variations are important for influencing inter-patient variability in drug response including cannabis (Hryhorowicz et al. 2018) (Hirota et al. 2013). Moreover, technology for pharmacogenetic testing has been made available to practitioners in frontline clinical settings. In Canada, commercial tests are available through several providers including GeneYouIn, Lobo Genetics and GeneXys. This availability, in combination with the pharmacist’s expertise in pharmacology and pharmacokinetics make them ideally suited to champion implementation of this novel technology to best optimize cannabis use by patients (American Society of Health-Systems Pharmacists n.d.).

The objective of this study is to evaluate the practicality and impact of implementing a pharmacogenetic screening program in the community pharmacy. This program is intended to help guide current or potential users of cannabis in both selection and pattern of use with the goal of minimizing potential adverse effects due to individual genetic variabilities. Pharmacogenomic testing involves identifying the polymorphism of key genes and pathways in cannabis metabolism (Hryhorowicz et al. 2018). This information can be used by pharmacists to more accurately predict the likelihood of short and long-term negative risks of cannabis use in individuals (Hryhorowicz et al. 2018). While both cannabinoids, THC and CBD, have reported therapeutic indications, specific genetic polymorphism have been demonstrated to be major determinants of an individual’s rate of cannabinoid metabolism, predisposition to the development of psychosis, and risk of cognitive and memory impairments (Hirota et al. 2013).

The primary metabolic pathway of THC metabolism involves CYP2C9 (Hirota et al. 2013). The cannabinoid is hydroxylated to its active form (OH-THC) that has significant psychotropic activity (Bland et al. 2020). It is then further oxidized by CYP2C9 to its carboxylic acid form (COOH-THC) which is pharmacologically inactive (Bland et al. 2020). Genetic epidemiology studies have shown 15–20% of the population have the CYP2C9*3 allele (Hirota et al. 2013). This variant of the gene results in a much slower conversation of THC to its non-psychoactive form when compared to the “normal” CYP2C9*1 allele variant (Hryhorowicz et al. 2018). Consequently, these individuals with *3 alleles can accumulate levels of THC that are 200–300% higher than those with the normal allele (Hirota et al. 2013) and, hence, may be more predisposed to THC’s negative psychoactive effects including anxiety, hallucinations, paranoia, rapid heart rate and panic attacks (Hirota et al. 2013). It is often these negative effects that lead to motor vehicle accidents, workplace injuries, and hospitalizations (Hirota et al. 2013; Hartman and Huestis 2013).

Furthermore, guidance from Health Canada and the National Institute on Health Abuse strongly suggest that polymorphisms of AKT1 and COMT genes can impact response to cannabis use and predict the potential risk of developing psychosis and cognitive impairment, respectively (Morgan et al. 2016; Radhakrishnan et al. 2014; Health Canada n.d.). AKT1 is a gene that encodes for protein kinase which is required for multiple cellular functions including dopamine signaling (Radhakrishnan et al. 2014).

Three case control studies (Van Winkel et al. 2011; Di Forti et al. 2012; Morgan et al. 2016) demonstrated that, depending on frequency of cannabis use, users with the AKT1 C/C genotype had between a two-fold (history of use) and seven-fold (daily use) increased risk in the development of psychosis compared to T/T carriers (Morgan et al. 2016; Radhakrishnan et al. 2014; Van Winkel et al. 2011; Di Forti et al. 2012). Van Winkel et al. 2011 examined cannabis induced cognitive alterations in psychotic disorder using standardized verbal memory and sustained attention testing measures in relation to ATKI variability (Van Winkel et al. 2011). Siblings or parents were used when possible as case controls. Di Forti et al. 2012 is a retrospective case-control study that recruited patients aged 18 to 65 years who presented with their first episode of psychosis. Over the same period, at same mental health sites, healthy control subjects with similar characteristics were recruited (Di Forti et al. 2012). Morgan et al. 2016 recruited healthy young cannabis users who were assessed with the Psychotomimetic States Inventory, a 48-item questionnaire, for schizotypal symptoms (Morgan et al. 2016). These studies strongly support using AKT1 as a genetic marker to assess the genetic predisposition for psychosis in cannabis users (Radhakrishnan et al. 2014).

COMTis a second gene of interest, which encodes for catechol-O-methyltransferase (COMT) (Henquet et al. 2006). This enzyme plays a critical role in dopamine degradation in the brain. Two double-blind, placebo-controlled studies (Henquet et al. 2006; Tunbridge et al. 2015) have reported that functional polymorphism in the COMT gene may increase the risk of the immediate neurocognitive impairments associated with cannabis use (Henquet et al. 2006; Tunbridge et al. 2015). There are two common variants of COMT (Val and Met) corresponding to high and low enzymatic activity, respectively (Henquet et al. 2006). Henquet et al. 2006 used a cross-over design enrolling patients with a psychotic disorder, relatives of patients with a psychotic disorder, and healthy controls who were exposed to THC or placebo. This followed a cognitive assessment and an evaluation of current psychotic experiences. This study determined that carriers of the COMT Val allele were more sensitive to THC’s memory and attention impairments compared to carriers of the Met allele. Tunbridge et al. 2015 assessed cognitive performance in participants without a psychiatric diagnosis, using a between-subjects design (THC vs. placebo) and administered the treatment intravenously. The results showed that COMT genotype moderates the cognitive, but not psychotic effects of acutely administered THC. These studies demonstrated that carriers of the Val allele were more sensitive to THC-induced cognitive, memory and attention impairments (Tunbridge et al. 2015). As such, polymorphisms in COMT can be used as a possible marker of cannabis induced neurocognitive impairments such as memory loss or poor reaction time (Tunbridge et al. 2015).

## Methods

The pilot program is designed as an open-label, non-randomized, and observational study. Two urban Shoppers Drug Mart community pharmacies in Toronto, Canada offered pharmacogenomic testing to current and potential cannabis users as part of their professional services programs over a period of two months. Participating pharmacists received structured, comprehensive training in pharmacogenetics. Training consisted of a combination of online learning modules and small group interactive sessions with clinical geneticists from Lobo Genetics®. Pharmacists then facilitated voluntary subject enrollment among patients who they felt may benefit from screening. Rationale for testing included current and potential users of cannabis and those who have had a past negative experience of cannabis use. Participants must be over 18 years of age and able to provide informed consent. Lobo Genetics® provided pharmacogenomic tests at no cost to the study participants.

Patients who enrolled in the study completed a pre-test questionnaire providing their demographic information, medical history and prior experience with cannabis. Patients who enrolled were either submitting a prescription for medical cannabis, had general questions on cannabis use or was discovered of a history of cannabis use through pharmacist consultation. Approximately 75% of eligible patients consent to the study. Those who declined had concerns over privacy or did not comprehend the rationale of such test. Enrolled patients received three buccal swabs using a DNA cheek swab kit. De-identified, barcoded samples were then sent by regular mail to an off-site CLIA-certified laboratory for analysis in Mississauga, Ontario, Canada. A pharmacogenetic testing platform from Lobo Genetics® was utilized for translation of participants’ DNA with respect to CYP2C9, AKT1 and COMT genetic polymorphisms. Following genomic data translation, a personalized, evidence-based report was generated. Within 24 to 48 h of receiving the genetic results, pharmacists provided a cannabis pharmacogenetic consultation to patients via telephone or in-person. During the consultation, the pharmacist would assess the participants’ current or potential use of cannabis and their potential risk for adverse effects. Strategies to mitigate the risk were also recommended by the pharmacist based on the individual’s genetic profile. Participants were then asked to complete a post-test questionnaire assessing the perceived value of both the pharmacogenetic testing and the pharmacist’s consultation on influencing the safe use of cannabis.

## Results

Twenty patients were enrolled in the study. Table 1 outlines the demographic characteristics of the patient population. Half of the participants were completely unaware of the THC content in the cannabis that they were using or had used. The top 3 reasons for cannabis use included anxiety/depression (70%), recreational (40%) and pain management (35%). A review of medication history revealed that 45% of the patients were taking at least 1 chronic medication and of those patients, all (100%) of them were on at least 1 psychiatric medication such as an antidepressant, antipsychotic or sedative.

**Table 1:** Cannabis use history of 20 medical cannabis patients in Toronto, Canada

Mean age (years)	47
Gender	65% female
Current cannabis users	
Recreational	10
Medical	4
Past cannabis users	6
Frequency of cannabis use	
Few times daily	1
Once daily	5
Few times/week	4
Few times/month	2
Few times/year	2
Duration of cannabis use (years)	
0–1	6
1–2	3
3–5	4
5+	7
Products used	
Inhalation (smoked or vaporized)	14
Edibles	4
Oil	3
Capsule	1
Purpose for use	
Anxiety/depression	14
Recreational	8
Pain	7
Insomnia	3
Nausea/vomiting	1
Chronic medications (n)	
0	11
1–4	9
Patients on psychiatric medication (n)	9

Patients who enrolled in the study completed a pre-test questionnaire providing their demographic information, medical history and prior experience with cannabis. These patients were recruited while they were either submitting a prescription for medical cannabis, had general questions on cannabis use or was discovered of a history of cannabis use through pharmacist consultation

**Fig. 1:**
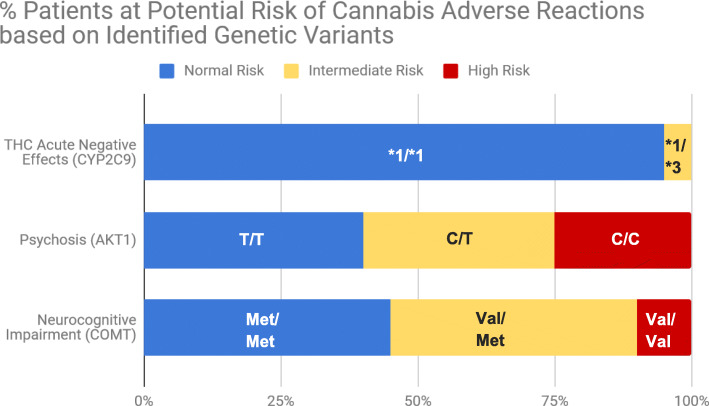
Pharmacogenetic screening identified 95% of participants having the CYP2C9*1/*1 genotype (suggesting normal THC metabolism), with 5% at potential risk of developing THC acute negative effects including anxiety, hallucinations, paranoia, rapid heart rate and panic attacks. Furthermore, 35 and 25% had AKT1 genotypes suggesting intermediate risk (C/T genotype) or high risk (C/C genotype), respectively, for cannabis-induced psychosis; and 45 and 10% had COMT genotypes suggesting intermediate risk (Val/Met genotype) or high risk (Val/Val genotype), respectively for cannabis-induced neurocognitive impairment

Consistent with epidemiological studies, pharmacogenetic screening identified that the majority of the study population (95%) had the CYP2C9*1/*1 genotype. All these patients would be expected to have normal THC metabolism (Fig. 1) without an increased risk of adverse reactions. Conversely, analysis of the AKT1 and COMT genotypes revealed that over half (> 55%) of the participants had gene variants that significantly increased their risk of potentially developing psychotic symptoms and/or cognitive impairment. More specifically, using AKTI as a genetic risk marker for developing psychosis, 35 and 25% of patients were identified as being at intermediate risk (C/T genotype) and high risk (C/C genotype), respectively. Similarly, testing of the COMT genotype revealed that 55% of participants were at elevated risk for neurocognitive impairment, with 45% having an intermediate risk (Val/Met genotype) and 10% at high risk (Val/Val genotype).

Table 2 summarizes the results of a post screening questionnaire. After receiving a pharmacist consultation reviewing their pharmacogenomic test results, 65% of patients stated that the interaction increased their overall comfort level in choosing a specific strength/strain of cannabis for use in the future. Also, 75% of participants reported a high value in this service and believed the testing might have significant impact on their future use of cannabis. Overwhelmingly, 95% of patients felt that pharmacists should be more involved in advising patients on the appropriate use of cannabis.

**Table 2:** Attitudes towards pharmacogenetic testing among 20 medical cannabis patients in Toronto, Canada

**1. Do you think you will change your cannabis usage due to the test results?**	
Stop completely	15%
Reduce use	5%
No change	75%
Increase use	5%
**2. Did the test results change your comfort level in choosing a specific strength/strain of cannabis?**	
Less comfortable	0%
No change	35%
More comfortable	65%
**3. Do you think pharmacists should be more involved in advising on cannabis use?**	
No	5%
Neutral	5%
Yes	90%
**4. What is the impact/value of the genetic results regarding cannabis use?**	
Low	10%
Neutral	15%
High	75%
**5. What is the impact/value of the pharmacist’s consult towards safe cannabis use?**	
Low	0%
Neutral	5%
High	95%

During the consultation, the pharmacist would assess the participants’ current or potential use of cannabis and their potential risk for adverse effects. Strategies to mitigate the risk were also recommended by the pharmacist based on the individual’s genetic profile. Participants were then asked to complete a post-test questionnaire assessing the perceived value of both the pharmacogenetic testing and the pharmacist’s consultation on influencing the safe use of cannabis with results shown

## Discussion

This is the first study of its kind to examine the use of pharmacogenomic testing to guide cannabis education in a community pharmacy setting. Our small pilot study was able to demonstrate that pharmacists could successfully implement pharmacogenetic testing and provide clinically significant information to patients in guiding cannabis use. Importantly, our findings suggest that patients highly valued the pharmacist consultation and being able to use personalized genetic information to make decisions about future cannabis use.

Surprisingly, given our small sample size, a number of clinically important genetic variations were identified by the program. Although the study did not find any CYP2C9 variants associated with highly diminished THC metabolism, analysis of the AKT1 and COMT genotypes revealed that over half (> 55%) of the participants had gene variants that significantly increased their risk of potentially developing psychotic symptoms and/or cognitive impairment. The high frequency of these cannabis-gene interactions is concerning as only 20% patients reported having their cannabis use monitored by a physician (Table 1).

For example, one study participant was referred to the pilot program for pharmacogenomic screening after a recent hospitalization due to a severe adverse event post cannabis use for their first time. Subsequent testing revealed that the patient is genetically predisposed as high risk for both psychosis (AKT1 C/C genotype) and cognitive impairment (COMT Val/Val genotype). This case illustrates the potential benefits of pharmacist-directed screening prior to initial cannabis selection and use. Genomic testing would have identified and potentially avoided the cannabis-gene interactions. Subsequent pharmacist consultation had led the participant to decide to completely abstain from cannabis use and if the decision were made to use cannabis in the future, a product of lower THC content would be selected due to their genetic predisposition for adverse effects.

Moreover, it is well documented that patients with psychiatric conditions frequently use cannabis as a means of managing their symptoms in addition to or in place of prescription medications or medical consult (Health Canada n.d.). Forty-five percent of the participants were taking chronic medications, in which all of them were on at least one psychiatric drug. Additionally, 70% of participants reported they were using cannabis in an attempt to self-manage their depression and/or anxiety. Cannabis-drug interactions can negatively impact the cannabis users’ experience (Health Canada n.d.). Even though cannabis-drug interactions are not well studied, caution must be taken when combining cannabis with psychiatric medications such as antidepressants, antipsychotics or sedatives, especially with the older classes, due to the inhibitory/inductive effects on drug metabolism and/or additive effects (University of Washington Alcohol and Drug Abuse Institute n.d.; Brown and Winterstein 2019; Alsherbiny and Li 2019). Given that almost half of the patients were taking psychiatric medications, this highlights the significant opportunity for pharmacists to be actively involved in identifying potential cannabis-gene and cannabis-drug interactions.

This initial study is limited by its small sample size and lack of specific quantified risk associated with the different genetic variants. The study addresses only participant satisfaction and attitudes of cannabis use, but did not follow up on actual behavioral changes or clinical outcomes. Future studies will aim to address these limitations.

## Conclusion

Although the study did not find any CYP2C9 variants associated with highly diminished THC metabolism, most of these patients do carry genetic variants that predisposes them to the potential development of psychosis and memory impairment, especially with higher concentrations and/or longer durations of THC use. Similar initiatives can potentially improve patient safety and empower individuals to make informed decisions about cannabis use and possible complications.

## Data Availability

N/A

## Abbreviations

C: Cysteine
CBD: Cannabidiol
CLIA: Certified laboratory for analysis
COMT: Catechol-O-methyltransferase
COOH-THC: Carboxy-tetrahydrocannabinol
CYP2C9: Cytochrome P450 2C9
DNA: Deoxyribonucleic acid
Met: Methionine
OH-THC: Hydroxy-tetrahydrocannabinol
THC: Tetrahydrocannabinol
Val: Valine
